# Supplementary material for: Comparative multiomics analysis of cell physiological state after culture in a basket bioreactor
Source: Sci Rep. 2022 Nov 23;12:20161. doi: 10.1038/s41598-022-24687-4 (PMC9686226; doi:10.1038/s41598-022-24687-4)
Supplement: Supplementary file 1 — Supplementary Information 1. [file 41598_2022_24687_MOESM1_ESM.zip › raw data/Transcriptomic raw data/7.AS/2.dasplot/TE1vsTC1/A3SS/15_CACNA2D1_NW_023666042.1_37058210_37058266_-@NW_023666042.1_37058210_37058281_-@NW_023666042.1_37066521_37066595_-.pdf]

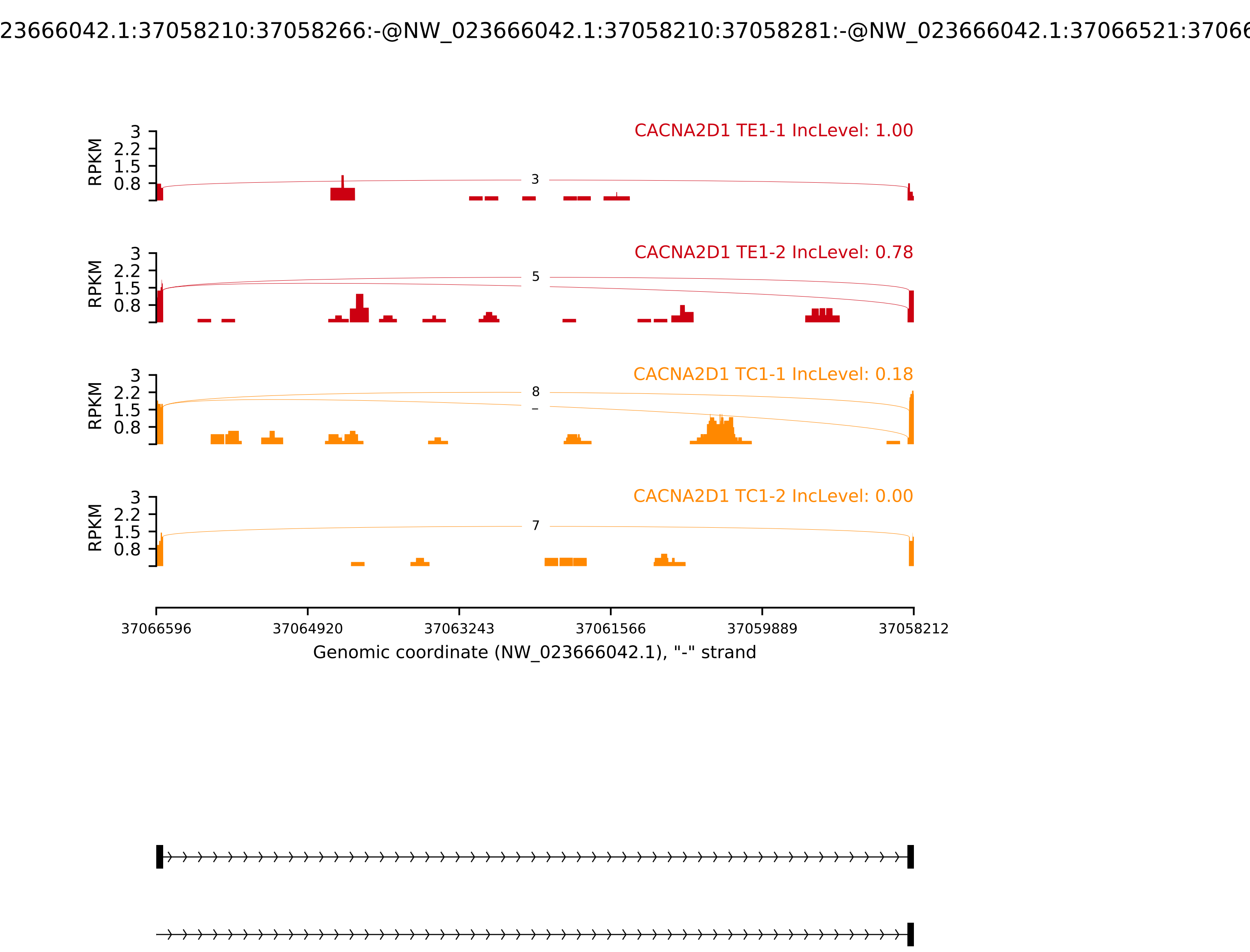
23666042.1:37058210:37058266:-@NW_023666042.1:37058210:37058281:-@NW_023666042.1:37066521:3706
CACNA2D1 TE1-1 IncLevel: 1.00
CACNA2D1 TE1-2 IncLevel: 0.78
CACNA2D1 TC1-1 IncLevel: 0.18
CACNA2D1 TC1-2 IncLevel: 0.00
RPKM
3
2.2
1.5
0.8
3
5
8
7
37066596
37064920
37063243
37061566
37059889
37058212
Genomic coordinate (NW_023666042.1), "-" strand
